# The Anatomical Position of the Caput Coli; Deviations from the Normal Type

**Published:** 1891-05

**Authors:** Thomas H. Manley

**Affiliations:** Visiting Surgeon to the Harlem Hospital, New York; 302 West Fifty-Third Street, New York


Buffalo Medical and Surgical Journal

Vol. XXX. MAY, 1891. No. 10.

### Original Communications.

### THE ANATOMICAL POSITION OF THE CAPUT COLI; DEVIATIONS FROM THE NORMAL TYPE.

By THOMAS H. MANLEY, M. D.

Visiting Surgeon to the Harlem Hospital, New York.

Where is the profession or craft so credulous or gullible as that of Medicine?

Let some one answer that if he can; and, then for the present, let us in a cursory way, direct attention to that disease of an inflammatory type, which arises in the tissues which enter into and are connected with the caput coli; besides, examine into those occasional vagaries of development, one of which is presented here.

At the inferior terminus of the spinal column, man has but the rudiment of a tail, which, however, is not free, but is attached to and is imbedded in muscular and other tissues. In the right iliac fossa we find the rudimentary cecum, a small tail or worm-shaped relic of the enormous pouch in which the final act of digestion is completed, in all the herbiverous mammalia. Its purpose in the economy of the human being is unknown, although it is often the seat of pathological changes, the etiology of which is not yet well understood. The caudal appendage, so-called, varies in length, and is usually in the peritoneal cavity. It is pervious, has usually an independent mesentery, a serous investment, a thick muscular coat, and its mucosa is continuous with the colon.

The caput coli is not a blind pouch, as its name implies (cecum) but is a direct continuation downward of the large intestine, and is entered at an angle, about two inches from its terminus or base, by the ilium, at which point we get the ileo-cecal portal. It normally may be regarded like the ascending and descending colon, the duodenum and rectum, as one of the fixed viscera, *i. e.*, it has like them a stationary immovable position and has *but a partial investment* of peritoneum. That it is but partly covered by the peritoneum is self-evident and incontestible, else it could not be a fixed viscus, for it is retained in position and intimately incorporated with the cellular tissues, along its posterior boundary.

Occasionally the caput coli has a long free mesentery, a mesocolon, when it is nearly wholly enclosed by the peritoneum and may slip down and through the inguinal canal, or make an excursion across to the left of the medium line, or in other directions.

Now, recently, particularly since the appearance of Frederick Treves's observations on the peritoneum were published, in which he took the ground that the cecum is *always wholly* covered by peritoneum, many surgeons maintain the same view and deny that it has any investment, except this serous membrane. Accordingly, in consonance with this view, we are taught that typhlitic abscess is always within the peritoneal cavity,—a doctrine which is as deceptive and dangerous as it is without foundation or support.

How can any one who has eyes and the use of them admit any such preposterous assumption, and especially he who has access to all the standard works on anatomy?

It would seem that the modern views and theories on localized inflammatory changes about the cecum were as irrational and inaccurate in many respects, as are the dicta relating to its structural anatomy at variance with all former teachings and as untenable. Looking on the caudal appendage as the *fons et origo* of these pathological changes, as a prophylactic and curative procedure it is snipped away at its root with the caput coli. Hence, inflammation involving the walls of the bowel itself, or the cellular tissues, are to be hereafter wholly ignored and we must have no more typhlitis. But, unfortunately for the theory, we do have every grade of inflammation, ulceration and suppuration in the adjacent areolar tissues when the appendix is entirely free from implication.

These iliac phlegmons, they say, are always intra-peritoneal, or outside the general peritoneal cavity. Now, as a matter of fact, particularly speaking from a surgical standpoint, what do we find when we open on to a perityphlitic abscess from behind? Immediately on reaching the suppurating mass, after dividing the fascia transversalis, we come on the cellular *investment* of the pyogenic membrane. Dividing this, we at once enter the abscess cavity. This may extend in every direction, upward behind the lumbar fascia, or downward into the pelvis; but the peritoneal cavity is yet untainted, unless the abscess-wall burst directly into it, which is seldom the case. Certainly the position and extent of the abscess will vary, according to the situation in which it develops ; but, even when the disease giving rise to it starts in the appendicular elements, it will invest it with an envelope of fibro-plastic substance and take on adhesions with the mural peritoneum, so densely and intimately as to preclude one from being able to isolate, the one from the other ; and from this time, and all time, as far as that goes, the serous laminæ of the compromised peritoneum *are* so altered organically that their physiological and histological functions are *no longer* obvious. The endothelial layer is permanently destroyed. Those pyogenic cysts, which so commonly wall in diseased appendices, always, singularly, take a backward and upward direction, pressing the lax muscular tissues away from the iliac fascia.

It is of vital importance, then, that this disposition of pyogenic formation in the right iliac fossa be clearly understood, or else the practitioner, operating for the first time, may inflict much needless injury ; whereas, in commencing the line of attack in the right direction, the peritoneal cavity is wholly avoided, the pus-sac can be effectually drained and less liability to hernia will follow, after cicatrisation of the wound.

### MARKED ANOMALIES IN THE POSITION OF THE APPENDIX.

In a recent report submitted by one of our deputy-coroners and the family physician of the deceased, in which a civil suit was instituted by the family to recover life-insurance, one of the doctors employed by the plaintiff showed me the following short paragraph which particularly attracted my notice. The doctors in their report said that : "Having opened the peritoneal cavity, nothing abnormal was noted until the cecum was exposed, when no trace whatever of the appendix could be seen, nor was there any evidence of scar or cicatrix." Although this statement is scarcely explicit enough, as it does not say absolutely that the appendix was wanting, yet one can scarcely put any other construction on the language employed.

On writing to my friend, Dr. Wm. O'Meagher, the deputy-coroner on duty at the time, he tells me that in his capacity as a military surgeon during the late war, and in forty years practice, during which time he made some six or seven hundred autopsies, he had never met with a similar case, and, as the appendix did not come into view in this instance, it was presumed absent. He said that the caput coli had a broad attachment posteriorly and was not disturbed. This, undoubtedly, explains the reason the caudal appendage was not found. Recently, in making an autopsy, I discovered that no appendix could be found by the most pains-taking search, in the peritoneal cavity. In order to positively assure myself that no oversight had been committed, I made a second transverse incision upon the superior spine of the ilium on the left, to the right side, thereby laying bare the entire cecum, yet no trace of the appendix could be seen. Now, gradually detaching carefully the cecum, which had a very broad cellular investment posteriorly, I came on the missing loop, the appendix, which was coiled on itself, imbedded in the cellular elements on the surface of the iliac fascia, entirely outside of and behind the peritoneal cavity. The appendix was four inches long and entirely pervious. There was no trace of inflammatory thickening, or any previously diseased condition in its vicinity. The cecum itself was of great capacity, being, when distended, more that nine inches in circumference, six inches in length, and nearly five inches in width. More than one-third of its surface was imbedded in the cellular tissues. The ascending colon, all the way up to the hepatic flexure, was of nearly double its normal dimensions and had a very broad cellular insertion.

Quain, Gray, Maglise, Wilson, Darling and Holden, with the French anatomists, Bichât, Cloquet, and Delpech, each and all say that the appendix usually, normally, lies free within the peritoneal cavity. Treves is the only author of distinction who claims that the cecum is wholly surrounded by peritoneum. It is accordingly noted that the singular situation of the cecum and the concealed position of the appendix, here reported, is quite unique. Since I made the dissection I have had a communication from Dr. W. M. Van Cott, of Brooklyn, N. Y., who tells me that, in making more than five hundred autopsies, he never encountered but a single case in which the caudal appendage was wholly behind and concealed by the posterior wall of the colon.

The surgical importance of this anomalous position of the appendix cannot be over-estimated when, through pathological changes, operative measures are imperative. Of this I was made aware practically, some time since, when I was called in consultation to see a case in which this arrangement obtained, the first time I ever met with it in the living subject.

The patient was a sailor, who, two years previously, in the Azores, had, he said, a very severe attack of "inflammation in the bowels," and thereafter, from time to time, noticed a thick flocculent matter in his urine, which always appeared at the end of the act of micturition.

Always after each recurrent attack of violent, colicy pains in his right groin this discharge would appear in considerable quantities, when he would experience marked relief.

From the commencement of the attack, which now confined him to bed, none had been seen in his urine, and to this fact he attributed the aggravation of his symptoms.

His physicians believing that there was an abscess formation near the cecum, invited me in to see him on the sixth day after the development of his latest attack. He then had high fever, with a quick pulse. There was total loss of appetite and great bodily weakness present. A distinct fulness could be seen and outlined over, and in the right iliac fossa. The abdomen was tender over its entire area.

I agreed with the medical attendant in diagnosis and advised an immediate operation. But the family would not consent until another practitioner saw the case. He regarded the localized fulness attributable to impaction and recommended abundant and often-repeated enemeta. His instructions were fully carried out, but without any benefit, and the following day I operated. Commencing the primary incision on a line parallel with Poupart's ligament, in about its center, I carried it upward about four inches and was soon within the peritoneal cavity, on the bare cecum. This was a little disappointing, for I expected to meet the abscess wall before the intestine was exposed. Now, being within the peritoneum, I sought for the appendix, but there was no trace of it, nor the presence of extensive adhesions, in which it might be imbedded. Just now one of the doctors present ventured that a mistake in diagnosis had been made. But, gently moving my fingers about, I detected fluctuation *behind* the cecum and made another incision farther backward and was gratified to find a large accumulation of matter, which issued up in increased quantities when pressure was made on any part of the abdominal surface, thus indicating how extensively the matter had penetrated through the retro-serous tissues. By widely separating the lips of the incision, the tip of the appendix could not be seen, and no attempt was made to remove it. With the aid of abundant irrigation and rigorous antisepsis, after an enormous mass of purulent matter, feces and *débris* of exposed tissues came away, the wound finally healed and he perfectly recovered.

Unless the general peritoneum is invaded and the appendix is opened, I never make an attempt to remove it, and in no instance in which I have operated, by free incision and drainage, has there been any relapse; nor do I regard an appendisectomy, at all, as a necessary measure when the pyogenic membrane thoroughly walls off the serous-cavity. After perforation it undoubtly cicatrizes, atrophies and is no longer a possible source of inconvenience or danger.

It may perhaps, in this connection, be permissible to cite from Hurd's essay (*Med. Age*, March, 1891,) who, quoting from Gottman, says: "that many mild cases of typhlitis are not diagnosticated at all, and that ninety-five per cent. get well. Gottman, in eleven years in the Moabit hospital, had ninety-six cases of iliac-phlegmon, and five only dying. There would have been as many deaths he says if they had all been operated on." This is carrying prejudice too far, yet any candid unbiased observer of the present, will, without hesitation, undoubtedly admit that the tide has turned the other way and we may often operate when there is no pressing necessity, and we may leave our patient worse off, than if, perhaps, nothing was done whatever by way of surgical interference.

302 West Fifty-Third Street, New York.

